# Manganese-containing polydopamine nanoparticles as theranostic agents for magnetic resonance imaging and photothermal/chemodynamic combined ferroptosis therapy treating gastric cancer

**DOI:** 10.1080/10717544.2022.2059124

**Published:** 2022-04-09

**Authors:** Zhian Chen, Zhenhao Li, Chuangji Li, Huilin Huang, Yingxin Ren, Zhenyuan Li, Yanfeng Hu, Weihong Guo

**Affiliations:** Department of General Surgery & Guangdong Provincial Key Laboratory of Precision Medicine for Gastrointestinal Tumor, Nanfang Hospital, Southern Medical University, Guangzhou, China

**Keywords:** Photothermal therapy, chemodynamic therapy, oxidative damage, ferroptosis

## Abstract

Gastric cancer (GC) is a serious disease with high morbidity and mortality rates worldwide. Chemotherapy plays a key role in GC treatment, while inevitable drug resistance and systematic side effects hinder its clinical application. Fenton chemistry-based chemodynamic therapy (CDT) has been used as a strategy for cancer ferroptosis, and the CDT efficiency could be enhanced by photothermal therapy (PTT). With the trend of treatment and diagnosis integration, the combination of magnetic resonance imaging (MRI) and CDT/PTT exhibits enormous progress. Herein, we constructed a platform based on PEGylated manganese-containing polydopamine (PDA) nanoparticles, named as PEG-PDA@Mn (PP@Mn) NPs. The PP@Mn NPs were stable and globular. Furthermore, they demonstrated near-infrared (NIR)-triggered PTT and Fenton-like reaction-based CDT effects and T1-weighted MRI capabilities. According to *in vitro* studies, the PP@Mn NPs trigger ferroptosis in cancer cells by producing abundant reactive oxygen species (ROS) via a Fenton-like reaction combined with PTT. Furthermore, *in vivo* studies showed that, under MRI guidance, the PP@Mn NPs combined with the PTT at the tumor region, have CDT anti-tumor effect. In conclusion, the PP@Mn NPs could provide an effective strategy for CDT/PTT synergistic ferroptosis therapy for GC.

## 1. Introduction

Gastric cancer (GC) is a common malignancy with high morbidity worldwide, especially in East Asia, and is a serious threat to human health (Erratum, 2020). In China, most patients with GC are diagnosed with advanced disease, in which case adjuvant chemotherapy is preferred for GC treatment. However, easy drug resistance and severe side effects limit the application of chemotherapy. Furthermore, in cancer therapy, chemical drugs as reactants are quickly consumed by interacting with pathological/pathogenic entities, eventually losing their effectiveness (Devita & Chu, 2008). To achieve a therapeutic effect, high and repeated doses are administered to maintain the drug concentrations, which causes side effects (Yang et al., 2019). Therefore, there is an urgent need to develop drug delivery systems (DDSs) with novel strategies to improve the efficacy and limit the side effects of tumor chemotherapy.

Patel et al. (2013) reported ferroptosis for the first time, which is different from apoptosis, necrosis, and other traditional modes of cell death. The main processes of ferroptosis are the iron-dependent Fenton reaction and the inactivation of glutathione peroxidase 4 (GPX4) protein ROS promoting cells, facilitating the production of lipid peroxide (LPO), thus destroying the cell integrity and causing cell death (Yang et al., 2014; Stockwell et al., 2017). Increased cell ferroptosis can significantly inhibit tumor growth and play an important synergistic role in chemodynamic therapy (CDT) (Chen et al., 2021). CDT is an ROS-mediated therapeutic strategy based on Fenton chemistry, which was first reported by Fenton & Jackson (1899), including iron-based Fenton reaction and non-iron-based Fenton-like reaction. Essentially, a large number of oxidative hydroxyl radicals (•OH) are generated by the Fenton or reaction Fenton-like reaction between H_2_O_2_ and ions such as Fe^2+^ and Mn^2+^. Increasing preclinical evidence indicates that inducing ferroptosis may be an effective treatment strategy for delaying acquired resistance to multiple drugs, such as lapatinib and erlotinib, and that ferroptosis inducers can also synergize with traditional drugs (e.g. cisplatin) (Liang et al., 2019; Chen et al., 2021). Therefore, ferroptosis has significant potential for anti-tumor drug design. In particular, with the increasing number of in-depth applications of nanomaterials in anti-tumor drugs, nanomaterials show unique advantages in inducing tumor ferroptosis through Fenton or Fenton-like reactions (Shen et al., 2018; Tang et al., 2021). Compared to normal tissue, nanomaterials penetrate more easily into tumor tissue and can be retained for a longer period through the enhanced permeability and retention (EPR) effect (Maeda, 2001). For instance, Huo et al. (2019) developed a nanocatalyst containing elemental iron that effectively triggers the *in situ* Fenton reaction and produces toxic ·OH under the acidic microenvironment in the tumor, inducing cellular ferroptosis. Wang et al. (2018) reported that the manganese–oxygen bonds of manganese-doped silicon nanoparticles undergo oxidation/reduction in a glutathione-rich tumor cell environment and induce ferroptosis through a Fenton-like reaction. In addition, the Mn^2+^ ions (products of the abovementioned reactions) can be used as T1-magnetic resonance imaging (MRI) contrast agents for tumor imaging (Wang et al., 2018). Therefore, manganese is a promising component of the DDSs for CDT and MRI to facilitate the dynamic observation of tumors for local precision treatment.

Although the CDT promotes ferroptosis and has a considerable anti-tumor effect, it is still far from satisfactory. In recent years, studies have demonstrated that the combination of CDT and PTT has improved anti-tumor effect with broad prospects (Jia et al., 2020; Jiang et al., 2021). According to the classical thermodynamic molecular collision theory, the catalytic efficiency of the Fenton reaction is temperature-regulated, thus increasing the temperature may enhance the efficiency of the Fenton reaction. Min et al. (2020) developed a graphene oxide-based iron sponge exhibiting enhanced anti-tumor effects through PTT and Fenton chemistry. The graphene oxide-based DDS previously developed by our team produces excessive ROS after near-infrared (NIR) radiation and induces significant anti-tumor effects in GC through a combination of chemical/photothermal/photodynamic therapy (Guo et al., 2021).

Polydopamine (PDA) has excellent NIR-triggered photothermal conversion efficiency that can be used in tumor PTT (Liu et al., 2020). Some studies explored applying PDA coating to magnetic Fe_3_O_4_ nanoparticles and gold nano delivery systems and achieved excellent tumor PTT effects (Han et al., 2019; Wang et al., 2021). In addition, the PDA catechol functional group exhibits excellent chelation with metal ions (Fe, Mn, Zn, etc.), binding stably binding with manganese (Zhao et al., 2016; Zhang et al., 2020; Ding et al., 2021). In addition, MRI can guide synergetic CDT/PTT which corresponds to the trend of treatment and diagnosis integration.

**Scheme 1.**
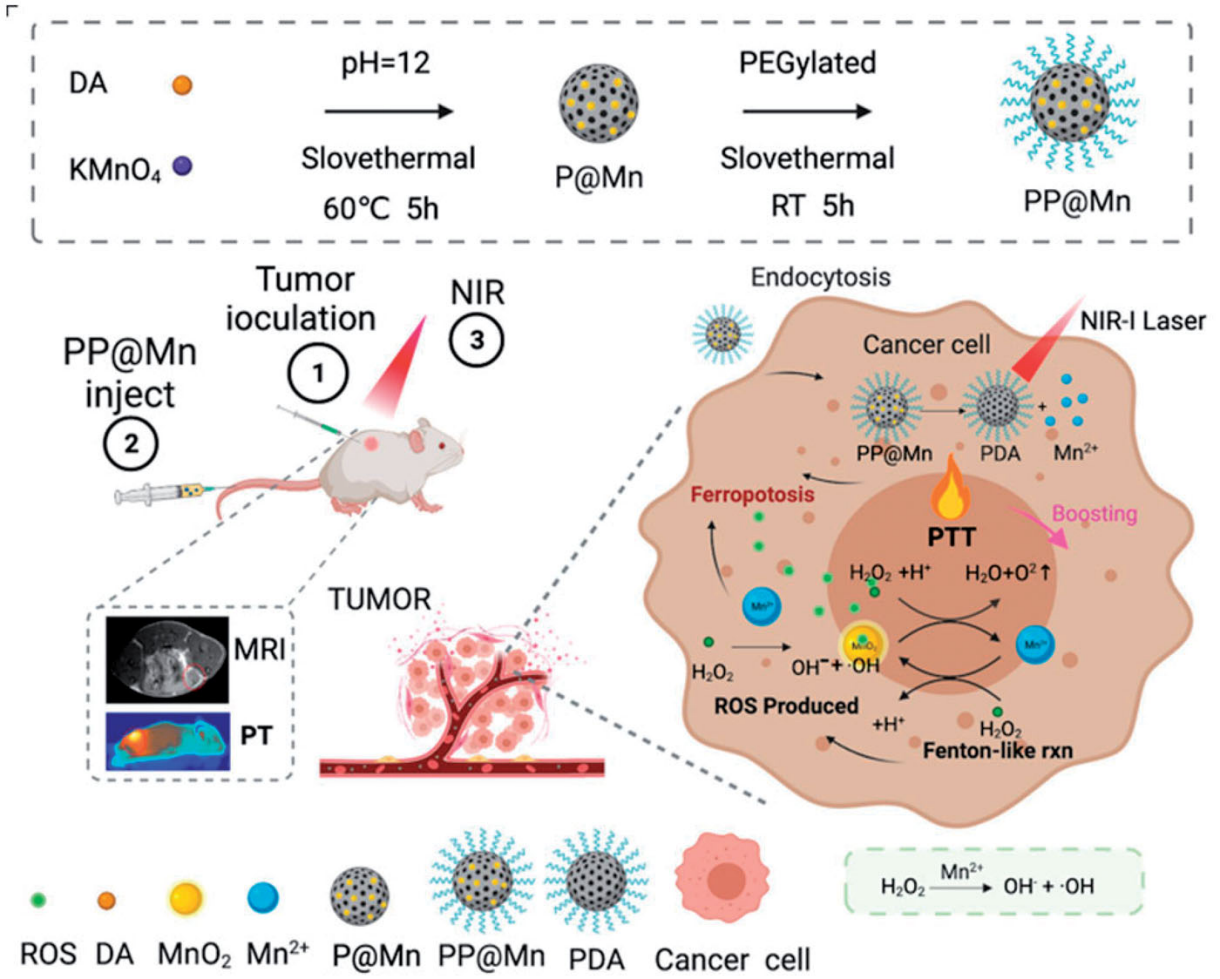
PP@Mn NPs enhanced tumor ferroptosis mediated by the Fenton-like reaction through mild photothermal effects guided by MRI. The prepared PP@Mn NPs accumulate in tumor tissues owing to the EPR effect and tumor MRI. After entering the tumor cells, the PP@Mn NPs decompose into the PDA and Mn^2+^ ions. PP@Mn NPs increase the temperature of the tumor site under NIR irradiation to achieve effective PTT. Moreover, the heat generated in the PTT process enhances the Fenton-like reaction, improving the production efficiency of ·OH, leading to ferroptosis.

In the present study, owing to the above-mentioned properties of PDA and manganese, the polyethylene glycol-modified (PEGylated) manganese-containing PDA nanoparticles (PEG-PDA@Mn [PP@Mn] NPs) were produced by a one-pot synthesis of potassium permanganate (KMnO_4_) with dopamine hydrochloride (Scheme 1). The obtained anti-tumor platform has the following advantages: (1) one-pot synthesis between PDA and KMnO_4_ could avoid complex synthesis modulation. (2) PP@Mn NPs combined with PTT enhance the Fenton-like reactions. (3) PP@Mn NPs play an anti-tumor role by promoting cellular ferroptosis. (4) MRI-guided tumor diagnosis and treatment integration. Combining mild PTT with CDT, the MRI-guided PP@Mn nanoplatform may play a therapeutic integration role as an effective ferroptosis therapy strategy in GC.

## 2. Experimental

### 2.1. Materials and reagents

Dopamine hydrochloride (98%) (Aladdin, Shanghai, China), KMnO_4_ (≥99.5%), Cell Counting Kit-8 (CCK-8) assay (Dojindo, Tokyo, Japan), reactive oxygen species (ROS) assay kit 2′,7′-dichlorofluorescin diacetate (DCFH-DA), C11-BODIPY (581/591) probe, rhodamine (Rho), phalloidin-FITC, LDH-cytotoxicity assay, and sodium hydrate (99%) were purchased from the Casmart and Rjmart platforms. PEG (NH_2_-PEG-NH_2_, MW 5000, 98%) were obtained from Guangzhou Tanshui Co., Ltd. (Guangzhou, China). Dulbecco’s minimum essential medium (DMEM), trypsin-containing ethylenediaminetetraacetic acid (EDTA), fetal bovine serum (FBS), phosphate-buffered saline (PBS), 4′,6-diamidino-2-phenylindole (DAPI) and penicillin-streptomycin solution were purchased from Thermo Fisher Scientific (Waltham, MA).

### 2.2. Preparation of PP@Mn NPs

To obtain the PDA NPs, NaOH solution (240 μL, 1 M) was rapidly added to the dopamine hydrochloride solution (99.90 mL, 10 mM, made with deionized water) in a round-bottomed flask. The solution gradually turned dark brown as the reaction proceeded under vigorous stirring at 60 °C for 5 h. To obtain the manganese-containing PDA (P@Mn) NPs, the NaOH solution (100 μL, 1 M) and KMnO_4_ solution (5 mL, 2 mM) were mixed and then rapidly added to the dopamine hydrochloride solution (94.90 mL, 10 mM) in a round-bottomed flask The solution quickly turned dark purple and then gradually turned deep black, and the reaction proceeded under vigorous stirring at 60 °C for 5 h. Polydopamine NPs and manganese-containing PDA NPs were collected by centrifuged (20,000 rpm, 15 min) and were washed thrice with deionized water to remove excess reactants. The products were freeze-drying to remove water and been redispersed in the deionized water. Twenty-four milligrams of NH_2_-PEG-NH_2_ was added to the PDA NP solution (10 mL, 2 mg/mL), and the pH of the solution was adjusted to 9 by NaOH solution (1 M). After vigorous stirring for 12 h at room temperature, the PEGylated PDA NPs were purified thrice by centrifugation (20,000 rpm, 15 min) with deionized water. The PP@Mn NPs were prepared using the same experimental procedure as for the PEGylation of the PDA NPs.

### 2.3. Characterization

The morphology and elements mapping of NPs were characterized by transmission electron microscopy (TEM) (JEOL JEM-2100F TEM, Tokyo, Japan). The chemical state and composition were characterized by X-ray photoelectron spectroscopy (XPS) (ESCALAB250Xi, Thermo Fisher, Waltham, MA). Electron spin resonance (ESR, JEOL, Ltd., Tokyo, Japan) was used to measure the production of •OH. The Mn content in the PP@Mn NPs was determined by inductively coupled plasma mass spectrometry (ICP-MS) (Optima7300DV, PerkinElmer, Waltham, MA). A Shimadzu UV-2600 UV-vis spectrophotometer (Kyoto, Japan) was used to acquire ultraviolet (UV)–vis absorption spectra. The hydrodynamic diameter and zeta potentials were measured using a Zetasizer Nano ZS (Malvern, Worcestershire, UK) by dynamic light scattering (DLS). The powder X-ray diffraction (XRD) patterns were acquired on a PANalytical X’Pert PRO X-ray diffractometer. The photothermal capability was assessed used an 808 nm semiconductor lasers (Shanghai Xilong Optoelectronics Technology Co., Ltd., Shanghai, China). Relaxivity of different PP@Mn NPs concentrations (0, 125, 250, 500, and 1000 μg/mL) was placed in tube holders for measurements by a 3.0-T Philips Achieva clinical MRI scanner (Philips Healthcare, Best, The Netherlands). The signal-to-noise ratio (SNR) was defined as: SNR = SI_mean_/SD_noise_. Infrared (IR) thermal images were acquired using an IR thermal camera (FLIR E50, Wilsonville, OR).

### 2.4. Fenton-like properties of PP@Mn NPs

The amount of •OH generated was analyzed using the classical colorimetric method, based on methylene blue (MB) degradation after selective •OH capture. Briefly, the absorbances at *λ* = 664 nm of the MB solution (10 μg/mL) in PBS (pH = 6.5) with 10 mM H_2_O_2_ were examined at 25 °C after adding PP@Mn NPs at different concentrations. The temperature was then increased to 42 °C for simulating the heat during PTT to evaluate the effect of temperature on the production of •OH.

### 2.5. Cell uptake and intracellular distribution of PP@Mn NPs

The mouse forestomach carcinoma (MFC) cells were collected and seeded into confocal dishes at a density of 1 × 10^5^ cells/cm^2^ for 24 h in DMEM medium contained 10% FBS. Then, the PDA and PP@Mn NPs labeled with Rho were added and co-incubated with the MFC cells for 1–8 h. Thereafter, the cells were fixed by 4% formaldehyde for 30 minutes, treated with 0.1% triton for 10 min, 5% BSA for 30 minutes, respectively, and stained with FITC-labeled phalloidin and DAPI. Finally, the uptake of the PDA and PP@Mn NPs in MFC cells was observed and captured using an Olympus FV3000 confocal laser scanning microscope (CLSM, Olympus, Tokyo, Japan).

### 2.6. Cytotoxicity assay

The MFC cells were seeded in 96-well plates at a density of 1 × 10^5^ cells/well and cultured for 24 h. Then, the cells were incubated with the PDA NPs and PP@Mn NPs. The photothermal groups (PDA NPs plus NIR, PP@Mn NPs plus NIR) were treated with NIR irradiation (808 nm, 0.75 W/cm^2^, 5 min). After 24 h, cell culture medium was removed. Then, 100 μL medium containing 10 μL CCK-8 regent was added to each well, and the cells were incubated for another 2 h. Finally, the cell viability was evaluated and recorded using a microplate reader (TecanSpark20m Multimode Microplate Reader, Männedorf, Switzerland).

Additionally, the LDH cytotoxicity assay kit was used to determine the cytotoxicity of the NPs. The MFC cells were seeded at approximately 5 × 10^3^ cells/well in each well of a 96-well plate and incubated overnight at 37 °C with 5% CO_2_. Subsequently, the cells were incubated with the PDA and PP@Mn NPs (with or without NIR irradiation), respectively. After 24 h, the medium was removed and the working solution was added in accordance to the manufacturer’s instructions, and the LDH activity was measured using the microplate reader.

### 2.7. Detection of intracellular ROS

To confirm intracellular ROS production, the MFC cells were seeded in 24-well assay plates and cultured for 24 h. Then, the PDA NPs or PP@Mn NPs were separately added to co-incubate with the MFC cells. The photothermal groups were treated with NIR irradiation (808 nm, 0.75 W/cm^2^, 5 min), and then all cells were cultured for 24 h. The suspension was discarded and supplemented with 1 mL of pure DMEM and incubated for an additional 20 min. The fluorescence signal of the cells was recorded at the same real-time exposure time (*λ*_ex_/*λ*_em_=488/520 nm). Fluorescence images were obtained using an inverted fluorescence microscope (Olympus, Tokyo, Japan).

### 2.8. Detection of lipid peroxidation

The MFC cells were seeded in a confocal dish and cultured for 24 h. Then, cells were cultured for 24 h after being treated with the control (PBS), PDA NPs, and PP@Mn NPs, with or without NIR irradiation (0.75 W/cm^2^, 5 min). To assess lipid peroxidation, the cells were incubated with C11-BODIPY (581/591) for 1 h and washed twice with PBS to remove excess dye. Subsequently, representative pictures were acquired by using the CLSM.

### 2.9. Western blot

The RIPA lysis buffer (Beyotime Biotechnology, Shanghai, China) was added into the treated tissue samples for extracting total proteins. Equal quantities of proteins were loaded onto a sodium dodecyl sulfate polyacrylamide gel electrophoresis (SDS-PAGE) gel and transferred onto polyvinylidene difluoride (PVDF) membranes (Millipore, Bedford, Germany). After probing with primary antibodies at 4 °C overnight, followed by blocking nonspecific antigens with 5% skimmed milk for 1 h, the membranes were subsequently washed and incubated with HRP-conjugated secondary antibodies. Finally, the ECL detection system (FDbio, Shenzhen, China) was used to visualize the signals.

### 2.10. Animal experiments

Female nude mice (age 4–5 weeks) were obtained from the Biomedical Research Institute of Southern Medical University (Guangzhou, China). Animal care and euthanasia were approved by the Institutional Animal Care and Use Committee (IACUC) of Southern Medical University (certification no. K2020015). We developed a tumor-bearing model by injecting the MFC cells, into the right flank of the female nude mice.

### 2.11. *In vivo* MRI experiments

After the GC tumor-bearing mice had been successfully constructed, the PP@Mn NPs were injected into the tail vein of the MFC tumor-bearing nude mice under anesthesia. Then, MRI under T1-weighted sequence (TR = 450.0 ms, TE = 15.3 ms, thickness = 2 mm) was performed and SNR was calculated.

### 2.12. *In vivo* anti-tumor effect of PP@Mn NPs

After the GC tumor-bearing mice had been successfully constructed, the MFC tumor-bearing nude mice were randomly divided into four groups (*n* = 5 in each group): (1) control (PBS), (2) PDA NPs plus NIR, (3) PP@Mn NPs, and (4) PP@Mn NPs plus NIR. Tumor volumes and body weights of the mice were measured every four days after the different treatments. On day 19, the mice were euthanized. The tumors in all groups were harvested and weighted. Additionally, the tumors were cut into tumor slices for immunofluorescence (IF, TUNEL, Abcam, Fremont, CA) and H&E staining. Tail vein injection dose: 100 μL and 2 mg/kg, at 7, 10, and 13 days after tumor-bearing. Irradiation: 808 nm laser, 0.75 W/cm^2^, and 5 min on tumor sites.

### 2.13. Statistical analysis

The mean ± standard deviation (SD) was used to analyze numerical values of the data. The IF intensity was quantitatively analyzed using ImageJ software (National Institutes of Health, Bethesda, MD). Unless otherwise stated, all the experiments were repeated at least three times. Unpaired Student's *t*-test or analysis of variance (ANOVA) was used, followed by Scheffe's post-test, and the data were analyzed numerically. Statistical significance was set at *p*<.05.

## 3. Results and discussion

### 3.1. Characterization of PP@Mn NPs

**Figure 1.**
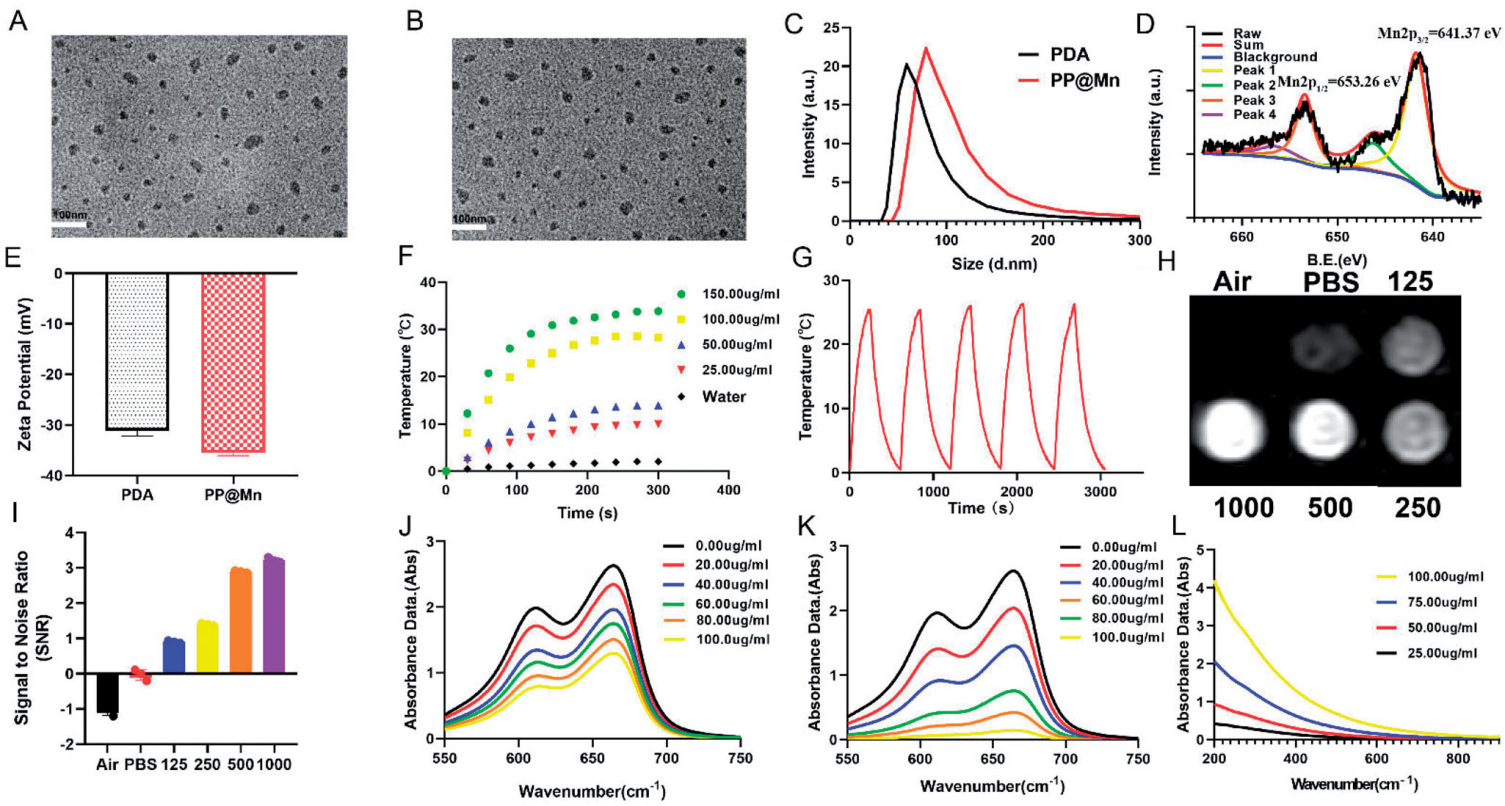
TEM images of PEG-PDA NPs (A) and PP@Mn NPs (B). (C) Hydrated particle sizes of PP@Mn NPs (*n* = 3). (D) XPS spectrum of PP@Mn NPs. (E) Zeta potential of PP@Mn NPs (*n* = 3). (F) Photothermal heating curves of control (PBS) and PP@Mn NPs in PBS at various concentrations under NIR irradiation (808 nm, 0.75 W/cm^2^). (G) Photothermal curves of PP@Mn NP solution (100 μg/mL) for five cycles under NIR irradiation (808 nm, 0.75 W/cm^2^, and 10 min). (H) *In vitro* T1-MRI of PP@Mn NP solution (0, 125, 250, 500, and 1000 µg/mL) was performed and the SNR value was calculated (I). UV–vis absorption spectra of the MB and H_2_O_2_ (10 mM) treated with different concentrations of PP@Mn NPs at 25 °C (J) or in a water bath at 42 °C (K). (L) UV–vis absorption spectra of PP@Mn NP solution.

The PP@Mn NPs were synthesized by a one-pot method through chemical oxidation polymerization of dopamine hydrochloride and KMnO_4_ under improved conditions (Liu et al., 2018). The reaction involves Mn chelation of eumelanin, accompanied by the reduction of KMnO_4_ (D'ischia et al., 2014). The TEM images (Figure 1(A,B)) revealed that the PDA NPs and PP@Mn NPs were well dispersed and exhibited a similar spherical morphology. As illustrated in Figure S1, the element mapping of PP@Mn NPs displayed that the coexistence of Mn, C, and O elements. Furthermore, from XRD results, PP@Mn NPs showed specific characteristic diffraction peak, while no Mn-related crystal was observed in PP@Mn NPs, indicating that Mn was doped in PP@Mn, but not as a crystal form (Figure S2, Supporting Information). DLS measurement showed that hydrodynamic size of the PDA NPs and PP@Mn NPs was 27.24 ± 1.01 nm and 36.39 ± 1.34 nm, respectively (Figure 1(C)). Meanwhile, PDI values of PDA NPs was 0.293 and that of PP@Mn NPs was 0.301. The zeta potentials of the PDA NPs and PP@Mn NPs were −31.13 ± 1.00 mV and −35.53 ± 0.52 mV, respectively, indicating the feasibility for biomedical applications (Figure 1(E)). XPS was performed to measure the valence states of Mn in the PP@Mn NPs (Figure 1(D)). The characteristic peaks centered at 641.37 and 653.26 eV were assigned to Mn 2p3/2 and Mn 2p1/2, respectively, suggesting the presence of a major portion of Mn (II) and a minor fraction of Mn (III) species. The content of Mn in the PP@Mn NPs was quantitatively analyzed by ICP-MS, determined to be 3.6 wt%. *In vitro* release of Mn from PP@Mn NPs was investigated by ICP-MS. A 20 μL sample of PP@Mn NPs (100 μg/mL) was immersed in PBS buffer with slight shaking. During 2 h, ∼31% of the Mn was released from the PP@Mn NPs. Furthermore, Mn release leveled off after 6 h and ∼42% of the Mn was released after 60 h (Figure S3, Supporting Information).

**Figure 2.**
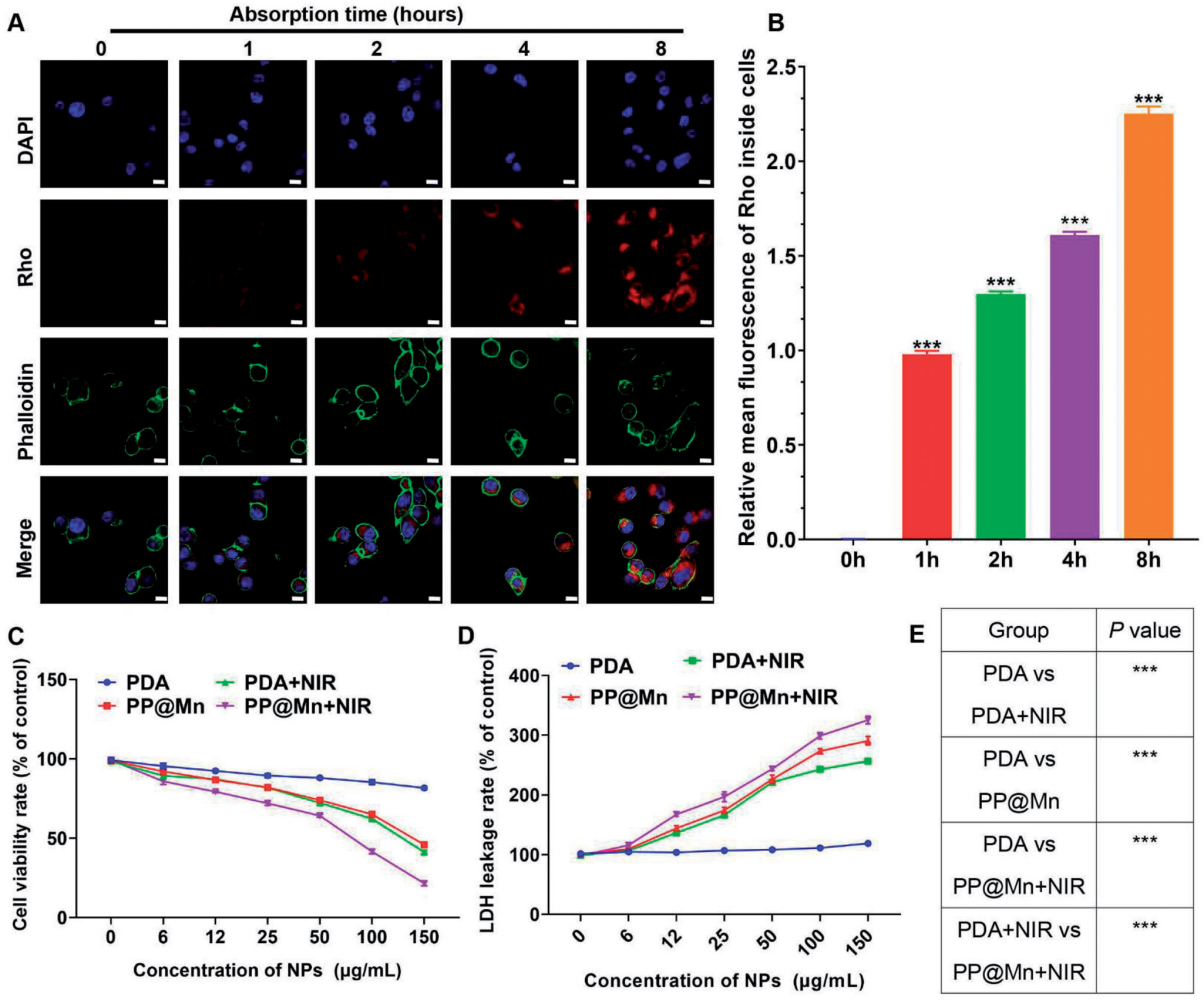
*In vitro* cellular uptake and anti-tumor effect of the PP@Mn NPs. (A) Representative images of MFC cells treated with Rho-labeled PP@Mn NPs for 1 to 8 h (left). Scale bar, 20 μm. (B) Relative mean fluorescence of Rho inside cells in (A). Relative cell viabilities (C) and relative LDH leakage (D) of MFC cells incubated with the PDA NPs and PP@Mn NPs with or without NIR irradiation (808 nm, 0.75 W/cm^2^, and 5 min) (*n* = 5). (E) *p* Value of (D).

**Figure 3.**
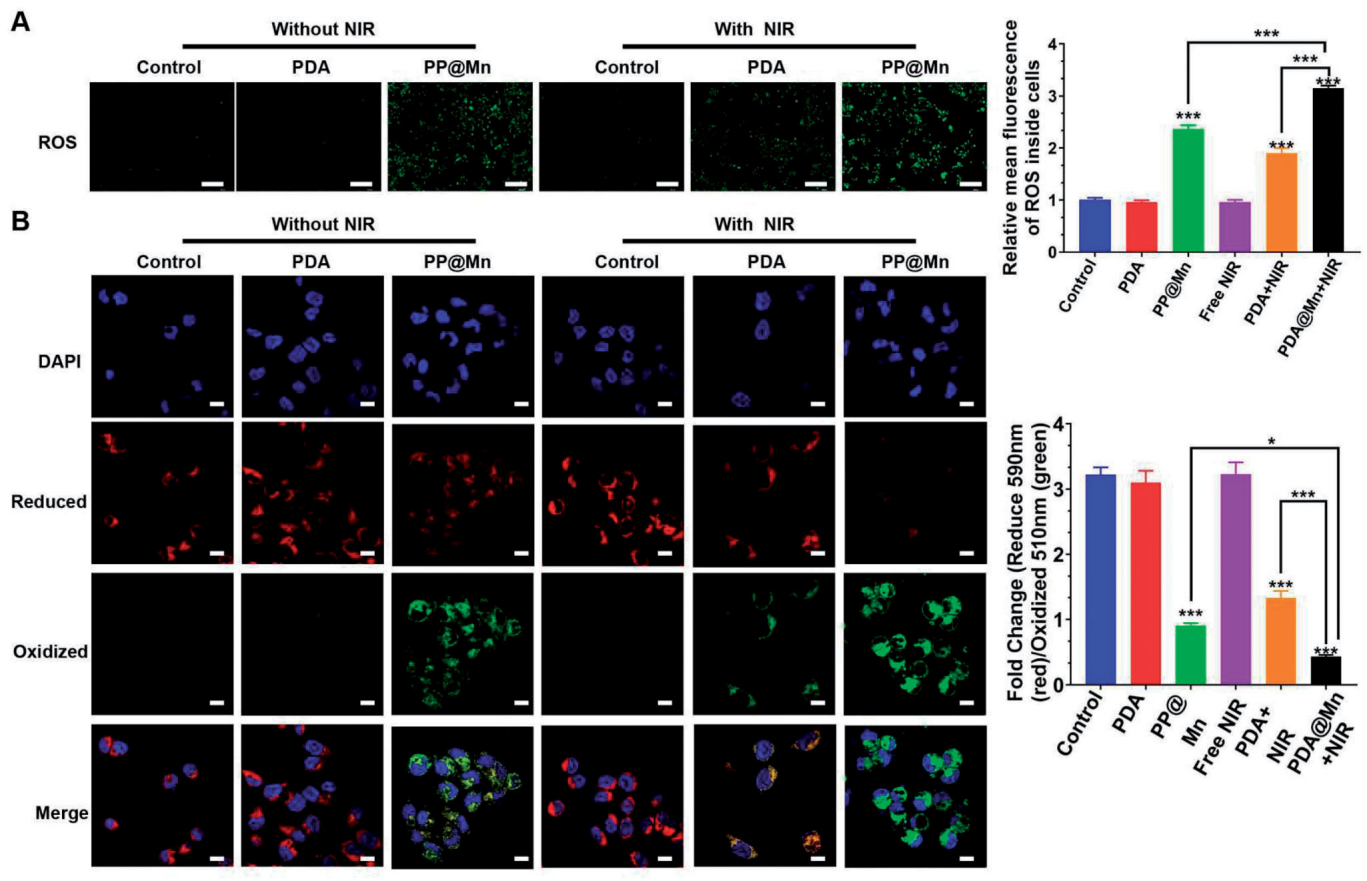
ROS production and ferroptosis level were verified. (A) Confocal images of MFC cells with different treatments using DCFH-DA as a ROS probe. ROS relative stress levels in different groups (*n* = 3). Scale bar, 100 µm. (B) Fluorescence confocal image of C11-BODIPY (581/591) probe detected lipid peroxidation. Scale bar, 10 µm.

To evaluate the photothermal performance of the PP@Mn NPs, photothermal heating curves of the PP@Mn NPs in PBS were recorded under NIR irradiation. The PP@Mn NP solution exhibited a concentration-dependent increase in temperature (Figure 1(F)). Notably, the temperature increment of the PP@Mn NP solution (150 μg/mL) reached approximately 32 °C, whereas that of the control group (PBS) was only 2 °C under the same experimental conditions. The photothermal consistency of the PP@Mn NPs is illustrated (Figure 1(G)). During five cycles of NIR irradiation, the stable responsiveness of the PP@Mn NP solution demonstrated the favorable photothermal stability of the PP@Mn NPs. Significant T1-MRI improvements revealed the concentration-dependent MRI ability of the PP@Mn NPs (Figures 1(H,I)). Owing to the relaxation properties of Mn^2+^ ions, the PP@Mn NPs are expected to be MRI contrast agents.

To determine the Fenton-like effect of the PP@Mn NPs, the produced ·OH was measured using a MB assay, which is based on MB degradation. As the concentration of the PP@Mn NPs increased, the MB absorption decreased, suggesting that the PP@Mn NPs produced abundant •OH (Figure 1(J)). The result was in accordance with previous report (Ou et al., 2021). Furthermore, to explore the influence of the photothermal effect on the Fenton-like reaction of the PP@Mn NPs, we placed the reaction systems in a water bath at 42 °C. The MB absorption of the PP@Mn NPs in a water bath at 42 °C was lower than those at 25 °C (Figure 1(K)). Approximately, a ninefold enhancement was observed in the PP@Mn NP solution(100 μg/mL) at 42 °C compared with that at 25 °C. This indicates that temperature increase improves the Fenton-like effect of PP@Mn NPs, inspiring us to further improve the efficacy of CDT by mild photothermal therapy (PTT). The generation of •OH was demonstrated by ESR and the typical 1:2:2:1 signal could be observed in the ESR spectrum. Furthermore, as illustrated in Supporting Figure 4, the generation of •OH was much more than other group, which was similar to the result of the MB degradation experiments. In addition, the UV–vis spectroscopy showed no special absorption bands for the PP@Mn NPs (Figure 1(L)).

**Figure 4.**
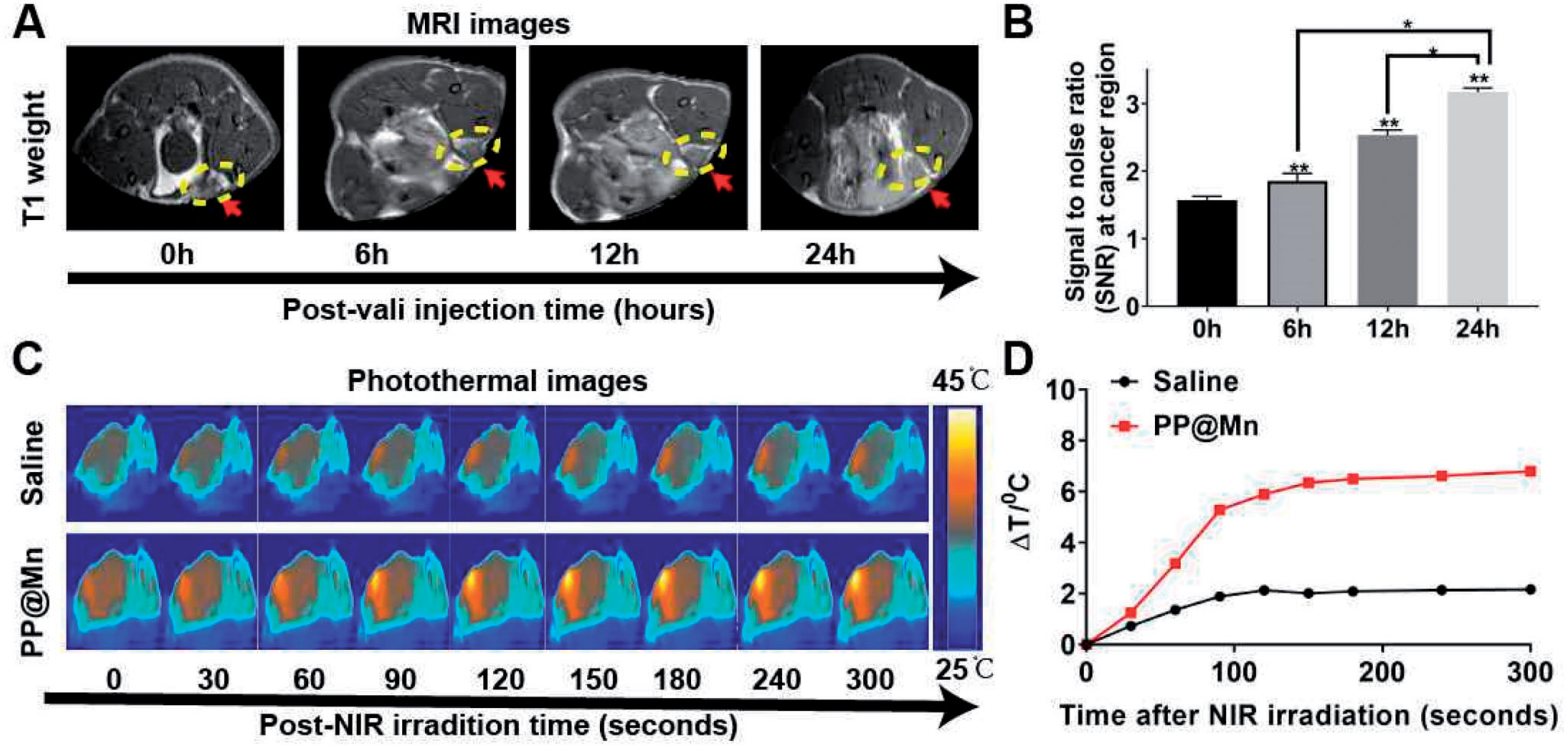
*In vivo* MRI and PTT of the PP@Mn NPs. (A) *In vivo* T1-MRI of MFC tumor-bearing mice after tail intravenous injection of PP@Mn NP solutions. (B) Quantitative analysis of MRI in (A). (C) Infrared thermal images of MFC tumor-bearing mice treated with PP@Mn NPs and saline under NIR irradiation. (D) The temperature changes against time of MFC tumor-bearing mice in (C).

### 3.2. *In vitro* cellular uptake and anti-tumor effect of PP@Mn NPs

The cellular uptake capacity of the PP@Mn NPs was determined. As shown in Figure 2(A), the MFC cells treated with Rho-marked PP@Mn NPs were labeled with phalloidin-FITC for cytoskeleton (green) and DAPI for nuclear DNA (blue) (Xu et al., 2020; Jiang et al., 2021). The fluorescent images of the cells were obtained by CLSM. For the absorption time of 4 h, some PP@Mn NPs were taken up by the MFC cells. For the absorption time of 8 h, the PP@Mn NPs occupied approximately the entire cytoplasmic space. The fluorescence intensity of cellular Rho was quantified (Figure 2(B)), showing that the cellular uptake of the PP@Mn NPs increased within 8 h. The Mn^2+^ in tumor cells can catalyze H_2_O_2_ to •OH through the Fenton-like reaction (Liu et al., 2020; Ou et al., 2021). Moreover, tumor PTT can elevate the •OH level while improving the efficiency of the Fenton-like reactions (Liu et al., 2020; Zhou et al., 2021). Therefore, the PP@Mn NPs were designed and applied to the combination therapy of CDT and PTT. To explore the possibility of application in cancer therapy, we evaluated the *in vitro* anti-tumor effects of the PDA and PP@Mn NPs with or without NIR irradiation. As shown in Figure 2(C), the MFC cell survival rate was all over 85% in the group exposed to the control group. The MFC cell survival rate in the PDA NPs plus NIR group decreased significantly as the concentration of PDA NPs increased, and the 50% inhibiting concentration (IC50) was approximately 130 μg/mL, suggesting that PTT contributed the most. The PP@Mn NPs also induced concentration-dependent cytotoxicity in the MFC cells, with an IC50 of approximately 140 μg/mL, due to the Fenton-like reaction. Moreover, the survival rate of the MFC cells was further reduced in the PP@Mn NPs plus NIR group, and the IC50 was approximately 75 μg/mL, indicating that introducing PTT enhanced the killing effect on cancer cells combined with the CDT. The LDH leakage is a biological marker of the cell damage caused by the disruption of the plasma membrane (Li et al., 2017). Therefore, cell injury was further assessed by the LDH assay. The highest LDH level was observed in the PP@Mn NPs plus NIR group, while the other three groups fit the trend of the cell viability assay (Figure 2(D)). These results indicate that the PP@Mn NPs can be efficiently taken up by cancer cells. Thus, the cancer cell killing capacity of PP@Mn NPs was significantly enhanced due to the considerable photothermal conversion property of the PDA and the CDT effect of the Mn^2+^. Therefore, the PP@Mn NPs demonstrated the potential for CDT/PTT combined cancer treatment.

The cellular uptake capacity of the PP@Mn NPs was determined. As shown in Figure 2(A), the MFC cells treated with Rho-marked PP@Mn NPs were labeled with phalloidin-FITC for cytoskeleton (green) and DAPI for nuclear DNA (blue) (Xu et al., 2020; Jiang et al., 2021). The fluorescent images of the cells were obtained by CLSM. For the absorption time of 4 h, some PP@Mn NPs were taken up by the MFC cells. For the absorption time of 8 h, the PP@Mn NPs occupied approximately the entire cytoplasmic space. The fluorescence intensity of cellular Rho was quantified (Figure 2(B)), showing that the cellular uptake of the PP@Mn NPs increased within 8 h. The Mn^2+^ in tumor cells can catalyze H_2_O_2_ to •OH through the Fenton-like reaction (Liu et al., 2020; Ou et al., 2021). Moreover, tumor PTT can elevate the •OH level while improving the efficiency of the Fenton-like reactions (Liu et al., 2020; Zhou et al., 2021). Therefore, the PP@Mn NPs were designed and applied to the combination therapy of CDT and PTT. To explore the possibility of application in cancer therapy, we evaluated the *in vitro* anti-tumor effects of the PDA and PP@Mn NPs with or without NIR irradiation. As shown in Figure 2(C), the MFC cell survival rate was all over 85% in the group exposed to the control group. The MFC cell survival rate in the PDA NPs plus NIR group decreased significantly as the concentration of PDA NPs increased, and the 50% inhibiting concentration (IC50) was approximately 130 μg/mL, suggesting that PTT contributed the most. The PP@Mn NPs also induced concentration-dependent cytotoxicity in the MFC cells, with an IC50 of approximately 140 μg/mL, due to the Fenton-like reaction. Moreover, the survival rate of the MFC cells was further reduced in the PP@Mn NPs plus NIR group, and the IC50 was approximately 75 μg/mL, indicating that introducing PTT enhanced the killing effect on cancer cells combined with the CDT. The LDH leakage is a biological marker of the cell damage caused by the disruption of the plasma membrane (Li et al., 2017). Therefore, cell injury was further assessed by the LDH assay. The highest LDH level was observed in the PP@Mn NPs plus NIR group, while the other three groups fit the trend of the cell viability assay (Figure 2(D)). These results indicate that the PP@Mn NPs can be efficiently taken up by cancer cells. Thus, the cancer cell killing capacity of PP@Mn NPs was significantly enhanced due to the considerable photothermal conversion property of the PDA and the CDT effect of the Mn^2+^. Therefore, the PP@Mn NPs demonstrated the potential for CDT/PTT combined cancer treatment.

### 3.3. *In vitro* CDT and PTT induced ferroptosis by PP@Mn NPs

Both the Fenton-like reaction and PTT produce ROS, enhancing the anticancer activity as a key factor in ferroptosis (Stockwell et al., 2017; Liu et al., 2019; Chen et al., 2021). The MFC cells were stained with the ROS-specific fluorescent probe DCFH-DA to monitor the ROS generation (Lin et al., 2020). The PP@Mn NPs reacted with H_2_O_2_ to form ROS through the Fenton-like reaction. As shown in Figure 3(A), the cells treated with the PP@Mn NPs under NIR irradiation exhibited the strongest intracellular green fluorescence, possibly due to the combined effects of Mn^2+^ and PDA. In contrast, rare green fluorescence was observed in the control, control plus NIR, and PDA NPs groups. After statistical analysis, the green fluorescence intensities in the PP@Mn NPs group was ∼2-fold higher than that in the control group. Thus, the CDT effect of Mn and the PTT effect of PDA enhanced the ROS production in the cancer cells. Notably, the PP@Mn NPs plus NIR group demonstrated approximately ∼3-fold ROS generation compared with that in the control group, indicating that the combination therapy with PTT and CDT significantly boosted ROS generation.

To detect the anti-tumor mechanism induced by the PP@Mn NPs, we analyzed the level of cell cytotoxic LPO, triggered by lipid peroxidation in cell ferroptosis (Yagoda et al., 2007). The C11-BODIPY probe was used to detect the level of LPO in the MFC cells treated with DMEM, PDA NPs, and PP@Mn NPs with or without NIR irradiation. As shown in Figure 3(B), the strongest green fluorescence was observed in the PP@Mn NPs group among the non-NIR groups, suggesting that extensive LPO generation occurs due to the CDT effect of Mn. In addition, the green fluorescence of the PP@Mn NPs plus NIR group and the PDA NPs plus NIR group was stronger than that of the free NIR treatment group, indicating considerable NIR effect of PDA for LPO generation. The MFC cells treated with the PP@Mn NPs plus NIR exhibited the strongest green and the least red fluorescence intensity, demonstrating the highest LPO level among all groups. As illustrated in Figure S5, compared with the control group, the expression of GPX4 protein in PP@Mn with or without NIR irradiation group was significantly decreased, but with NIR irradiation group showing the lowest expression. From these results, we inferred that the PP@Mn NPs significantly inhibited the proliferation of tumor cells by combining CDT with PTT. On the one hand, the PP@Mn NPs exhibited the Fenton-like reaction and PTT effect, producing abundant ROS, increasing the level of cellular oxidative stress, which induced ferroptosis in the tumor cells. On the other hand, PTT enhanced the Fenton-like response. Therefore, the rationally designed system achieved efficient anti-tumor activity through mild PTT synergistic chemotherapy, which may provide an experimental basis for the application of the PP@Mn NPs as anti-tumor drugs in combination therapies.

**Figure 5.**
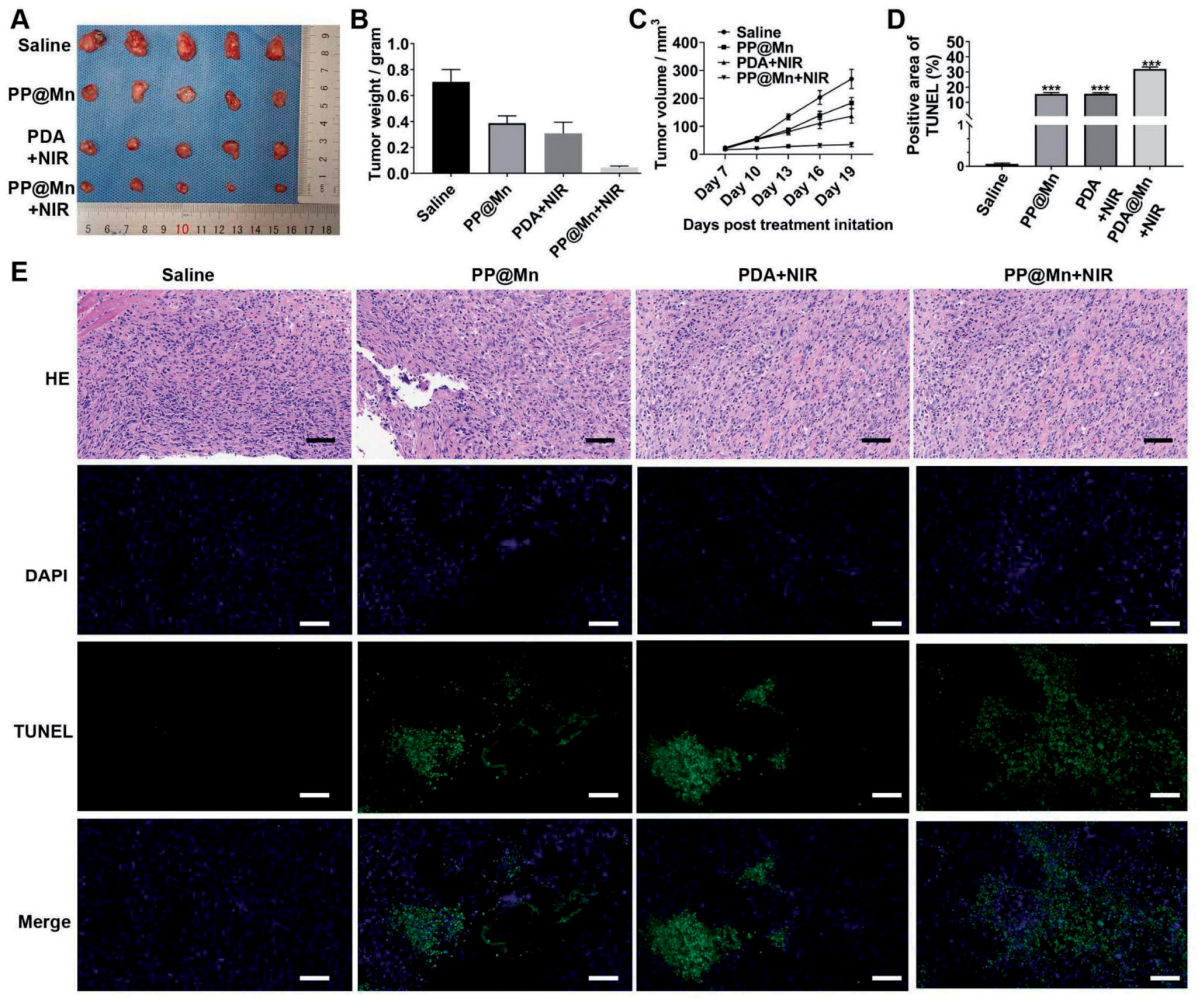
*In vivo* anti-tumor effect. (A) Photos of MFC tumor in different treatment groups. Weights (B) and volume (C) of MFC tumor in different treatment groups (*n* = 5). (D) Quantitative analysis of TUNEL staining images. (E) H&E and TUNEL staining images of tumor tissues excised from mice in different treatment groups. Scale bars, 50 μm.

### 3.4. *In vivo* MRI and PTT of PP@Mn NPs

The integration of cancer diagnosis and treatment has become a trend, while the MRI-guided PTT has considerable advantages. MRI can provide an accurate assessment of the time window for nanodrug accumulation in tumors, allowing for the precise administration of local PTT of the tumor. In particular, the Mn^2+^ has been widely used as a contrast agent for MRI (Ding et al., 2020; Liu et al., 2020). The PDA has been widely applied to the photothermal imaging and PTT of tumors owing to its excellent photothermal absorption capacity (Liu et al., 2018, 2020).

Here, the PP@Mn NPs were used as T1-MRI contrast agents for *in vivo* MRI. The MFC tumor-bearing mice were treated with a tail vein injection of the PP@Mn NPs at a dosage of 2 mg/kg, followed by T1-MRI. The signal intensity of MRI at the tumor site was enhanced 6 h after injection compared with that before injection (Figure 4(A)). The MRI signal intensity gradually increased over time, indicating that the PP@Mn NPs produce a significantly effective time-dependent MRI signal. The quantitative analysis of MRI confirmed the enhancement effect and further confirmed the effective accumulation of the PP@Mn NPs at the tumor site (Figure 4(B)).

Subsequently, the tumor sites of the photothermal groups were irradiated with NIR irradiation. We found that gradual warming could be achieved locally in the tumor, forming bright spots on the thermal image. After 5 min of irradiation, the tumors injected with the PP@Mn NPs were heated up by 6 °C, indicating that mild photothermal effects could be achieved with the PP@Mn NPs (Figure 4(C)). Together, these results demonstrate that our PP@Mn NPs can be used as a contrast agent for MRI and can be applied to mild PTT.

### 3.5. *In vivo* anti-tumor effect of PP@Mn NPs

Owing to the favorable *in vitro* cancer therapeutic effect of the PP@Mn NPs, *in vivo* anti-tumor efficacy was assessed in the MFC tumor-bearing mice (Figure 5(A)). When the tumors reached 20 mm^3^, the MFC tumor-bearing mice were randomly divided into four groups for a 19-day observation period. Compared with the control (PBS) group, the tumor growth in the PDA NPs plus NIR, PP@Mn NPs, and PP@Mn NPs plus NIR groups was partially inhibited (Figure 5(B,C)). The tumor growth inhibition of the PP@Mn NPs plus NIR group was significantly better than that of the PDA NPs plus NIR and PP@Mn NPs groups, demonstrating that PP@Mn NPs plus NIR had an excellent anti-tumor effect through CDT combined with mild PTT. On day 19, all mice were euthanized, and the tumors were excised. As shown in Figure 5, the tumors in the PP@Mn NPs plus NIR group were significantly smaller than those in the other three groups, with significant statistical differences. The tumor volumes in each group were quantitatively analyzed. The tumor weight of the PP@Mn NPs plus NIR group was the lowest among the four groups (Figure 5(B,C)). Hematoxylin and eosin (H&E) staining was performed to confirm the therapeutic effects of the different treatments. As shown in Figure 5(E), the most severe necrosis and deformed nucleus (karyopyknosis, karyorrhexis, and karyolysis) of cancer cells were observed in the tumor slices from the PP@Mn NPs plus NIR group among all groups.

Furthermore, the TUNEL assay revealed that apoptotic cells stained dark brown in the PP@Mn NPs plus NIR group were much less than those in the control group. Nineteen days after starting various therapies, the mice's major organs were also collected. The tissue sections showed no evident histological alterations, indicating negligible long-term adverse toxicity. These results showed that the PP@Mn NPs were highly effective in cancer therapy through CDT and PTT.

## 4. Conclusions

Herein, we constructed an MRI-guided PP@Mn NP theranostic nanoplatform, which induced ferroptosis in GC cells via combination therapy with mild PTT and CDT effects. Based on the *in vitro* experiments, the PP@Mn NPs generated abundant ROS through the Fenton-like reaction to damage cancer cells and the photothermal effect triggered by PDA significantly improved the Fenton reaction efficiency. The PP@Mn NPs were hypothesized to promote oxidative stress and induce ferroptosis in cancer cells. From the *in vivo* experiments, the PP@Mn NPs provided valuable tumor MRI visualization. Furthermore, the PP@Mn NPs significantly inhibited tumor growth and demonstrated satisfactory bio-safety. Hence, the PP@Mn NP nanoplatform represents a novel theranostic system for the MRI-guided CDT/PTT combined therapy, and needs further exploration for applications of ferroptosis-inducing nanomaterials in various fields.

## Data Availability

The datasets used during the present study are available from the corresponding author upon reasonable request.
