# Correction Notice

**DOI:** 10.1080/10717544.2022.2114992

**Published:** 2022-08-22

**Article title**: Manganese-containing polydopamine nanoparticles as theranostic agents for magnetic resonance imaging and photothermal/chemodynamic combined ferroptosis therapy treating gastric cancer

**Authors**: Zhian Chen, Zhenhao Li, Chuangji Li, Huilin Huang, Yingxin Ren, Zhenyuan Li, Yanfeng Hu and Weihong Guo

**Journal**: DRUG DELIVERY

**Bibliometrics:** Volume 29, Number 1, pages 1201–1211

DOI: https://doi.org/10.1080/10717544.2022.2059124

As per author’s request, figures 5 have been replaced in the published article.

